# Biomimetic Chromatography/QSAR Investigations in Modeling Properties Influencing the Biological Efficacy of Phenoxyacetic Acid-Derived Congeners

**DOI:** 10.3390/molecules30030688

**Published:** 2025-02-04

**Authors:** Małgorzata Janicka, Małgorzata Sztanke, Krzysztof Sztanke

**Affiliations:** 1 Department of Physical Chemistry, Faculty of Chemistry, Institute of Chemical Science, Maria Curie-Skłodowska University, Maria Curie-Skłodowska Sq. 2, 20-031 Lublin, Poland; malgorzata.janicka@mail.umcs.pl; 2 Department of Medical Chemistry, Medical University of Lublin, 4A Chodźki Street, 20-093 Lublin, Poland; malgorzata.sztanke@umlub.pl; 3 Laboratory of Bioorganic Compounds Synthesis and Analysis, Medical University of Lublin, 4A Chodźki Street, 20-093 Lublin, Poland

**Keywords:** biomimetic chromatography, biopartitioning micellar chromatography, immobilized artificial membrane chromatography, QSARs, hemolytic activity, phenoxyacetic acid-derived congeners

## Abstract

A hybrid method—combining liquid biomimetic chromatography techniques (immobilized artificial membrane chromatography and biopartitioning micellar chromatography) and Quantitative Structure–Activity Relationships—was used to derive helpful models for predicting selected biological properties such as penetration through the plant cuticle, the skin and the blood–brain barrier, and binding to human serum albumin of phenoxyacetic acid-derived congeners regarded as potential herbicides. Reliable, high-concept models were developed indicating the lipophilicity, polarizability, and sum of hydrogen bond donors and acceptors as properties that determine the biological efficacy of the title compounds. These models were validated by leave-one-out cross-validation. Modeling the toxicity of phenoxyacetic acid-derived congeners to red blood cells allowed the identification of the most toxic substances as well as those molecular descriptors that determine their hemolytic properties.

## 1. Introduction

The commercially available phenoxyacetic acid herbicides are widely used to control broadleaf weeds in agriculture, forestry, urban areas, and home gardens. Due to the progressive chemicalization of the environment, which affects the health of humans and animals, there is no doubt that understanding the physicochemical properties influencing the toxicity and effectiveness of this class of herbicides is fully justified [1,2].

Twenty-six mono-, di-, and trisubstituted phenoxyacetic acid-derived congeners (**1**–**16**, **18**, **21**–**29**) as synthetic auxins with potential herbicidal properties, as well as three commonly used herbicides (**17**, **19**, and **20**) (Table 1), were chosen in the current study. Because they mimic the physiological effects of the naturally occurring plant hormone heteroauxin, i.e., 2-(1*H*-indol-3-yl)acetic acid (which regulates the plant growth), their interaction with auxin-binding proteins is able to provoke the uncontrolled growth of weeds, which ultimately leads to their death [2]. Three herbicides from this class, i.e., 2-(2,4-dichlorophenoxy)acetic acid (2,4-D) (**19**), 2-(2,4,5-trichlorophenoxy)acetic acid (2,4,5-T) (**20**), and 2-(4-chloro-2-methylphenoxy)acetic acid (CMPA) (**17**)—with the highest auxinic activity even in low concentrations—have already been used in agriculture for weed control as plant protection products [2,3,4,5,6]. These ones belong to the third and fourth toxicity class, and their acute toxicity to the vast majority of experimental animals, expressed as the LD_50_ value, is in the range of 300–1000 mg kg^−1^ b.w. [7]. However, dogs are the most susceptible animals to this class of herbicides [8]. For them, the LD_50_ value of 2,4-D and 2,4,5-T is of 100 mg kg^−1^ b.w. [7]. Moreover, these chlorinated phenoxyacetic acid-derived molecules are classified as harmful for aquatic organisms [8]. Some studies have shown that 2,4-D induces genotoxic effects in vitro [5], whereas CMPA is not able to induce mutagenicity or genotoxicity in vivo [9]. According to the International Agency for Research on Cancer, 2,4-D has been classified as a possibly carcinogenic herbicide for humans (Group 2B) [10].

It was proven that the solubility and slightly acidic nature of commercially available herbicides from this class affects their uptake and movement within plant tissues [11]. There is no doubt that the use of herbicides in the active acid form must be controlled because their residues can be found in soil and groundwater, which may pose a threat to the health of humans and animals. As both three commercially useful herbicides (**17**, **19**, **20**) and the remaining twenty-six herbicide-like molecules (**1**–**16**, **18**, **21**–**29**) with the anticipated auxinic activity are or may be useful for weed control as plant protection products, their lipophilic and toxic properties as well as pharmacokinetic/pharmacodynamic data need to be assessed at the earliest stage of their development.

The use of liquid chromatography to predict the behavior of organic molecules in biological partitioning systems is a direct consequence of Colander’s findings leading to the formulation of rectilinear relationships known as Colander-type equations [12], i.e.:

log P_1_ = a_1_log P_2_ + b_1_ (1)

where P_1_ and P_2_ are the partition coefficients in the two different systems and a_1_ and b_1_ are the rectilinear regression coefficients.

Given the above and the definition of the retention factor k:

k = Kφ(2)

where K is the partition coefficient of the chromatographed substance between the stationary and mobile phases and φ is the phase ratio, i.e., the ratio of the mass (or volume) of the stationary phase to the volume of the mobile phase in a given chromatographic system, the following equation is true:

log P = a_2_log k + b_2_ (3)

where a_2_ and b_2_ are regression coefficients.

Equation (3) is the theoretical basis for using chromatographically measured (or determined) retention parameters to assess the lipophilic properties, i.e., partition coefficients in the *n*-octanol/water system (log P_o/w_), and also, especially in the case of biomimetic chromatographic systems, to predict the behavior of organic compounds in biological partition systems [13]. In the latter case, modern liquid chromatography proposes different techniques that mimic biological partitioning systems. These are techniques offering specific stationary phases, such as the immobilized artificial membrane (IAM) [14], stationary phases with immobilized proteins [15], cholesterol [16], etc., or mobile phases similar in composition to body fluids, such as micellar mobile phases [17]. A special case of the latter is biopartitioning micellar chromatography (BMC) using the non-ionic surfactant Brij 35 [18].

The prediction of biological parameters of new chemical entities considered as potential herbicides involves the search for quantitative relationships between the biological properties of a compound and the parameters characterizing its molecule, i.e., the implementation of Quantitative Structure Activity Relationships (QSARs) methodology. The result of this approach is the creation of hybrid models, which combine the in vitro chromatographic techniques with the in silico determined structural descriptors of molecules. This is an important aspect of screening for new bioactive substances, as it allows experimental verification of the calculated data.

The phenoxyacetic acid-derived congeners (**1**–**16**, **18**, **21**–**29**) identified in Table 1 as potential herbicides should be assessed for their ability to penetrate through the plant cuticle, which is a prerequisite for inducing the desired effect on weeds. At the same time, it is necessary to assess what potential risks the use of these compounds may pose to the environment, especially to other living organisms. Potential risks arising from the use of new herbicides should be assessed at the earliest possible stage of research to eliminate those congeners that do not show good promise and for which further testing is not advisable. Thus, it is important to predict their penetration through other biological barriers, such as the blood–brain barrier or skin, and binding to human serum albumin, on which the distribution in the animal body depends. Thus, in the present study, the parameters such as log P_w/pc_ (describing the partitioning of solutes in the water/plant cuticle system), log K_p_ (describing the permeation of solutes through the skin), log P_w/HSA_ (describing the partitioning of solutes in the water/human serum albumin system), and log BB (describing the permeation of solutes through the blood/brain barrier) were modeled.

The purpose of our studies is to use liquid biomimetic chromatographic techniques (such as biopartitioning micellar chromatography or immobilized artificial membrane chromatography) in combination with the QSAR methodology in modeling the indicated above properties of twenty-nine diversified phenoxyacetic acid-derived congeners, including three commonly used herbicides, that influence their effectiveness as potential herbicides. The subsequent goal is to evaluate, in experimental studies, their hemolytic effect on red blood cells, which is a good measure of their toxicity. To the best of our knowledge, the hemolytic properties of most of the twenty-nine tested compounds (especially herbicide-like molecules) are not known because they have not been tested so far. However, studies on 2,4-D have shown the correlation between its toxicity and interaction with the lipid bilayer of the erythrocyte membrane [3].

## 2. Results and Discussion

### 2.1. Toxicity of Phenoxyacetic Acid-Derived Congeners (***1**–**29***) on Red Blood Cells

To evaluate the toxicity of phenoxyacetic acid-derived congeners **1**–**29** on the most numerous cells in the blood, i.e., erythrocytes, a hemolysis assay was performed. The degree of hemolysis (calculated as the percentage of hemolysis) was assessed after incubation of red blood cells with a solution of each herbicide or herbicide-like compound at one of the ten concentrations tested (250, 500, 750, 1000, 1250, 1500, 1750, 2000, 3000 and 4000 µM). It was found that all the investigated molecules induced hemolysis, and their hemolytic activities were concentration- and structure-dependent, as shown in Figure S1 in the Supplementary Material. Based on these results, the lowest concentration causing 50% hemolysis (HC_50_) was calculated for each congener (Figure S2 in the Supplementary Material). The HC_50_ values for all the investigated molecules ranged from 1078 µM to 1982 µM (Table 2). The most toxic for erythrocytes proved to be compounds **7**, **25**, **24**, **27**, **9**, and **8**, with the HC_50_ values of 1078, 1124, 1136, 1188, 1190, and 1205 µM, respectively. It is seen that molecules **7**, **8**, and **9** bear the methoxy group in positions *ortho*, *meta*, and *para*, respectively, while **24**, **25**, and **27** contain the phenolic hydroxyl, alcoholic hydroxymethyl, and formyl group, respectively, in the phenoxy formation. In turn, the least toxic were compounds **12**, **14**, and **11** with 2,4-di-*tert*-pentyl, 2,4-dibromo, and 4-*tert*-butyl group, respectively, in the phenoxy moiety, for which the concentration causing 50% hemolysis was 1982, 1878, and 1826 µM, respectively.

According to literature data, phenoxyherbicides may have an adverse effect on erythrocytes. They induced the generation of reactive oxygen species (which led to oxidative damage to important cellular components), increased the peroxidation of lipids and proteins of the cell membrane, oxidized hemoglobin, affected the activity of antioxidant enzymes, and changed the morphology of red blood cells [19,20,21,22,23,24]. These harmful effects may lead to disruption of the integrity of the erythrocyte membrane and, consequently, to hemolysis.

One of the factors leading to hemolysis may be lipid peroxidation. A relationship between the intensity of this process and the type of substituent at the phenoxy formation of the approved auxin herbicide was observed [22]. Bioactive compounds substituted only with chlorine atoms, i.e., 2,4-D and 2,4,5-T, caused greater lipid peroxidation than the herbicidal molecule substituted with both chloro and methyl groups, i.e., CMPA. Simultaneously, the herbicide with two chlorine atoms (2,4-D) increased lipid peroxidation to a greater extent than the one with three chlorine atoms (2,4,5-T). The observed lipid peroxidation in the case of compounds with the chlorine substituent/substituents may result from the direct generation of free radicals by these molecules or from secondary processes, i.e., the formation of free oxygen radicals by oxyhemoglobin released in the process of erythrocyte hemolysis.

Additionally, 2,4-D may affect the structure of the erythrocyte membrane. X-ray diffraction studies have shown the interaction of this herbicide with dimyristoylphosphatidylcholine present in the outer lipid monolayer of the erythrocyte membrane. This may lead to the loss of the integrity of the erythrocyte membrane directly or indirectly by facilitating the penetration of lipid peroxidation-causing factors into the bilayer. Scanning electron microscopy has shown that, as a result of massive incorporation of 2,4-D into the outer lipid monolayer of the cell membrane, erythrocytes changed their shape from discoidal to echinocytic [24].

### 2.2. In Silico Characteristics

An assessment of the expected biological activity of the test compounds, including the potential environmental hazard, can be made on the basis of the parameters determined in silico (Table 3), using known rules: the rather general rule of five (Ro5) formulated by Lipiński [25], with more detailed information provided by Ghose et al. [26] or Clark [27] on the size of the molar masses, as well as van de Waterbeemd et al. [28] and Kelder et al. [29] on the topological polar surface area (TPSA) values. The numbers of hydrogen bond donors and acceptors are also taken into account as suggested by the studies of Krajl et al. [30], van der Waterbeemd [28], and Ajay et al. [31] concerning compounds that activate the central nervous system.

The analyzed phenoxyacetic acid-derived congeners are compounds with relatively low molecular weights (MW), ranging from 166.17 g mol^−1^ to 292.41 g mol^−1^, giving an average value of 208.99 g mol^−1^. They are also moderately lipophilic, as indicated by their log P_o/w_ values ranging from 0.424 to 3.379, with the exception of the highly lipophilic compound **12**, for which the log P_o/w_ = 6.535. The TPSA values are in the range of 46.53–95.36 Å^2^ and the polarizability (α) is in the range of 16.26–33.76 Å^3^. The number of hydrogen bond donors (HBD) is between one and two, the number of hydrogen bonds acceptors (HBA) is between three and six, and the number of rotatable bonds (NRB) is between three and seven. These values indicate that the above congeners fulfill the requirements for drug-like molecules and could therefore pose a real risk to humans and animals in the event of unintentional entry into the environment and further into animal organisms. The in silico log BB values (BB = concentration in brain/concentration in blood) are between −0.956 and 0.149, indicating that they are unlikely to penetrate from blood to brain, with the exception of molecule **12**, for which the log BB = 0.956. The log P_w/HSA_ values are between −0.735 and 0.673, indicating that these congeners should not bind too strongly to human serum albumin, again with the exception of compound **12**, for which the P_w/HSA_ value is 1.741. The log P_w/pc_ values between 0.343 and 6.201 indicate fairly good penetration of phenoxyacetic acid-derived congeners through the plant cuticle, with the exception of molecules **24** and **25**, for which the P_w/pc_ values are −0.08 and 0, respectively. These data are in favor of considering the vast majority of the above compounds as potential herbicides.

### 2.3. Chromatographic Data

The experimental chromatographic data, such as log k_BMC_ and log k_w,IAM_ (Table 3), measured in the two chromatographic systems used, correlate well with each other:

log k_BMC_ = 0.437(±0.020)log k_w,IAM_ + 0.684(±0.027); SD = 0.1062, R^2^ = 0.9600; R^2^_adj_ = 0.9589, R^2^_pred_ = 0.9534 (4)

confirming that both chromatographic parameters characterize the test compounds in an analogous way. The log k_w,IAM_ parameters correlate well with the in silico determined partitioning parameters, log P_o/w_:

log P_o/w_ = 2.010(±0.101) + 1.439(±0.137) log k_w,IAM_; SD = 0.5406, R^2^ = 0.8038; R^2^_adj_ = 0.7965, R^2^_pred_ = 0.7256 (5)

A slightly weaker correlation was obtained for the micellar parameters, log k_BMC_:

log P_o/w_ = 1.138(±0.152) + 2.007(±0.215)log k_BMC_; SD = 0.5944, R^2^ = 0.7627; R^2^_adj_ = 0.7539, R^2^_pred_ = 0.6785 (6)

where SD is the standard deviation, and R^2^, R^2^_adj_, and R^2^_pred_ are the values of determination coefficient R-squared, adjusted R-squared, and predictable R-squared, respectively. The above relationships allow us to consider these, especially the retention coefficients obtained on the IAM column, as good descriptors of the lipophilicity of the studied solutes.

### 2.4. Establishment of Quantitative Structure Property Relationships

The concept of Quantitative Structure–Activity Relationships is to specify quantitative relationships between the specific property of a solute (SP—solute property), which could be pharmacokinetic properties or toxicity, and its molecular descriptors. The essence of this approach is well illustrated by the following mathematical model [17]:

SP = f(lipophilic, electronic and steric properties)(7)

The search for a model for QSPRs is limited to the specification of a mathematical form, which is usually determined as an effect of multiple linear regression (MLR) [32]. The essence of the model search is to specify the independent variables to be included in the model, respecting the principle of orthogonality and minimizing the number of independent variables. The most commonly used descriptors describing the steric properties of a compound include molecular weight, molar volume, polarizability, and parachor. The number of rotatable bonds provides information about the flexibility and indirectly also about the size of the molecule [33]. The topological polar surface area and the number of hydrogen bond donors and acceptors are electronic descriptors [34]. The basic parameter characterizing the lipophilicity of the molecule is the partition coefficient in the *n*-octanol/water system, log P_o/w_ or, alternatively, the chromatographic lipophilicity descriptor (log k) [35].

As already mentioned, the independent variables (structural descriptors) used in the model cannot be related to each other. This can be ensured by a similarity analysis, e.g., by principal component analysis (PCA), a dendrogram, or the variance inflation factor (VIF). The derived models should be evaluated, e.g., using standard statistical methods; the coefficient of determination (R^2^) allows the evaluation of the fit of the obtained model to the output data. The adjusted R-squared (R^2^_adj_) and the predicted R-squared (R^2^_pred_) are used to compare the fit of regression models containing a different number of independent variables to assess the reliability of the predictions of the resulting model, respectively: the mean squared error (MSE) to assess the predictive ability and accuracy of the model (small MSE values result in more reliable predictions) and the sum of squares of the predicted residual error (PRESS) as a good estimate of the actual prediction error of the model (the smaller the PRESS value, the better the predictive ability of the model). In addition, QSPR models should be validated. In our study, leave-out cross-validation was used, which is frequently employed for this purpose [36,37].

**Figure 1:**
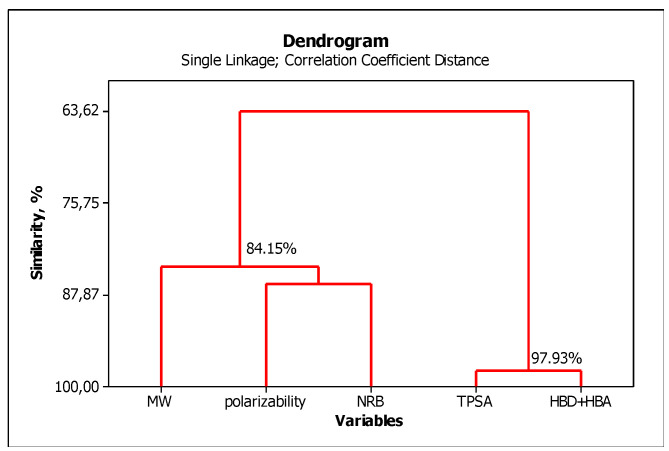
Similarities between molecular descriptors of the tested compounds.

In the study presented here, the structural descriptors listed in Table 3 were considered as independent variables, and the similarities between them were assessed using cluster analysis, as shown in the dendrogram (Figure 1). The variables considered form two clusters: the first shows 97.93% similarity in the parameters characterizing the electronic properties, i.e., TPSA and (HBD + HBA), and the second reveals 84.15% similarity in the parameters characterizing the molecular structure, i.e., MW, polarizability, and NRB. It is clear that the number of rotatable bonds influences both the mass of the molecule and its polarizability. The topological polar surface area depends on both the number of hydrogen bond donors and acceptors.

Using multiple regression and considering all possible combinations of uncorrelated structural descriptors and the chromatographic lipophilicity parameter (log k_BMC_ or log k_w,IAM_) (Table 3), we derived 24 models of QSARs from which the most promising models (M1–M8) were selected. These models are listed in Table 4 and the corresponding statistical data in Table 5.

**Figure 2:**
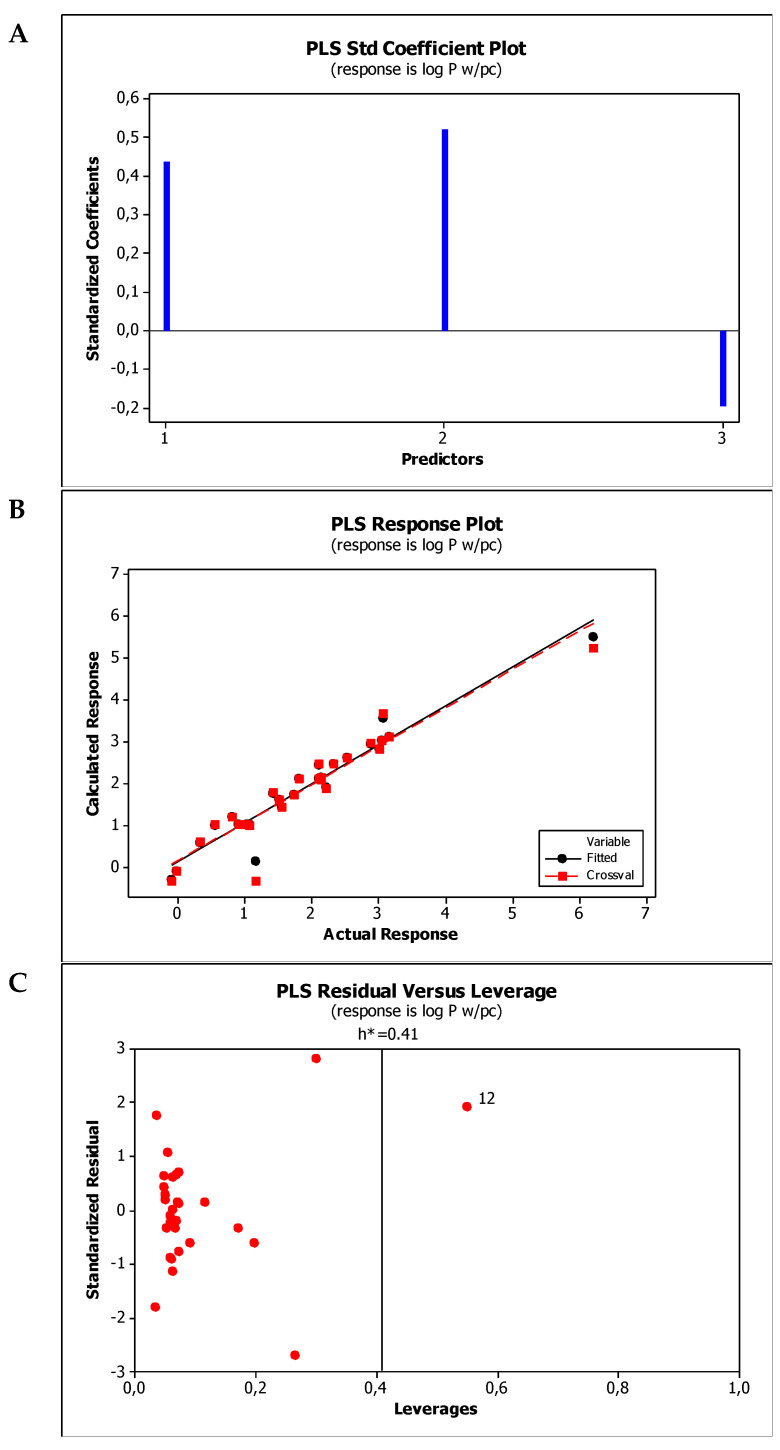
Standardized coefficients (**A**), response plot (**B**), and Williams plot (**C**) obtained for log P_w/pc_ prediction (model M1).

**Figure 3:**
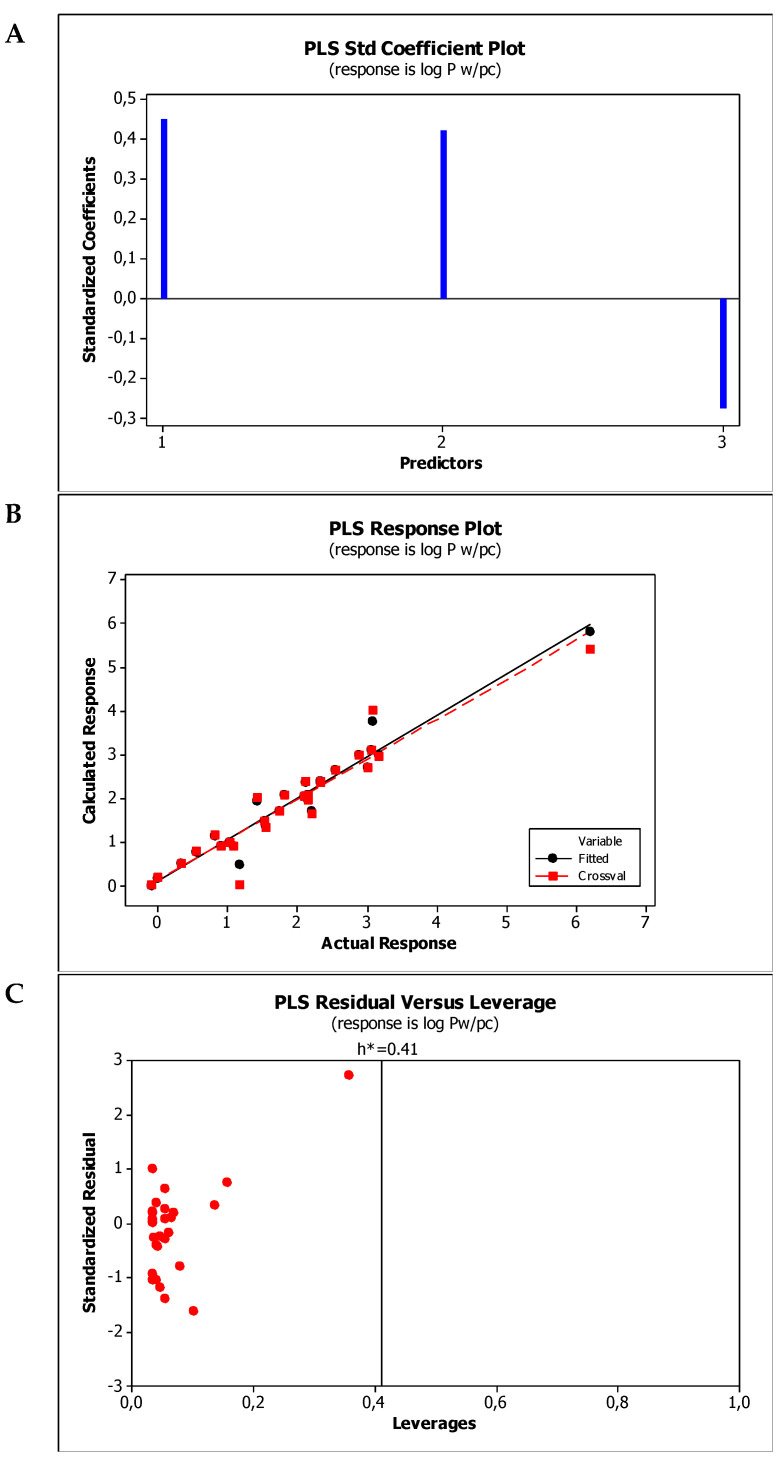
Standardized coefficients (**A**), response plot (**B**), and Williams plot (**C**) obtained for log P_w/pc_ prediction (model M2).

The derived models were cross-validated (leave-one-out). This involves evaluating the actual predictive power of a particular model in order to make a final decision on the best model. The results of the validation are shown in Figure 2 and Figure 3, and Figures S3–S6 in the Supplementary Material, and are based on the statistical parameters listed in Table 5. QSPR models are only valid in the applicability domain (AD), i.e., for the domain in which they have been validated [37]. AD is a space of (physico-chemical) information for which the model was developed and for which it is applicable to make predictions for new compounds. In the present work, the leverage approach (Williams plot) was used, where the warning leverage, h*, was calculated as follows:

h* = 3(p + 1)/n(8)

where n is the total number of samples and p is the number of descriptors involved in the correlation.

All statistical data allow us to evaluate the derived models as reliable and highly predictive; all values of the coefficient of determination R^2^ are significantly higher than 0.8, especially in the case of models M1, M2, and M4 [38]. The values of R^2^_pred_ are moderate for models M3, M4, M5, M7, and M8, and very good for models M1, M2, and M6. Also, in relation to the available literature data, these models should be considered very good and highly predictive. As reported by Faramarzi et al. [39], the R^2^ values of various models predicting organic compounds’ permeation through the blood–brain barrier vary from 0.54 to 0.99. Different QSAR models derived by Zeng et al. [40] to predict the skin permeability of various organic compounds show R^2^ values in the range of 0.483–0.901, and R^2^_adj_ values in the range of 0.481–0.899. Xue et al. [41] compared QSAR models to predict compounds’ binding affinities to human serum albumin and obtained R^2^ parameters varying in a wide range of 0.76–0.94. Singh et al. [42] and Gupta et al. [43] report robust models for predicting penetration through the plant cuticle, for which R^2^ values are in the ranges of 0.903–0.923 and 0.956–0.976, respectively. There is no information available in the literature on the R^2^_pred_ values of the QSAR models.

**Figure 4:**
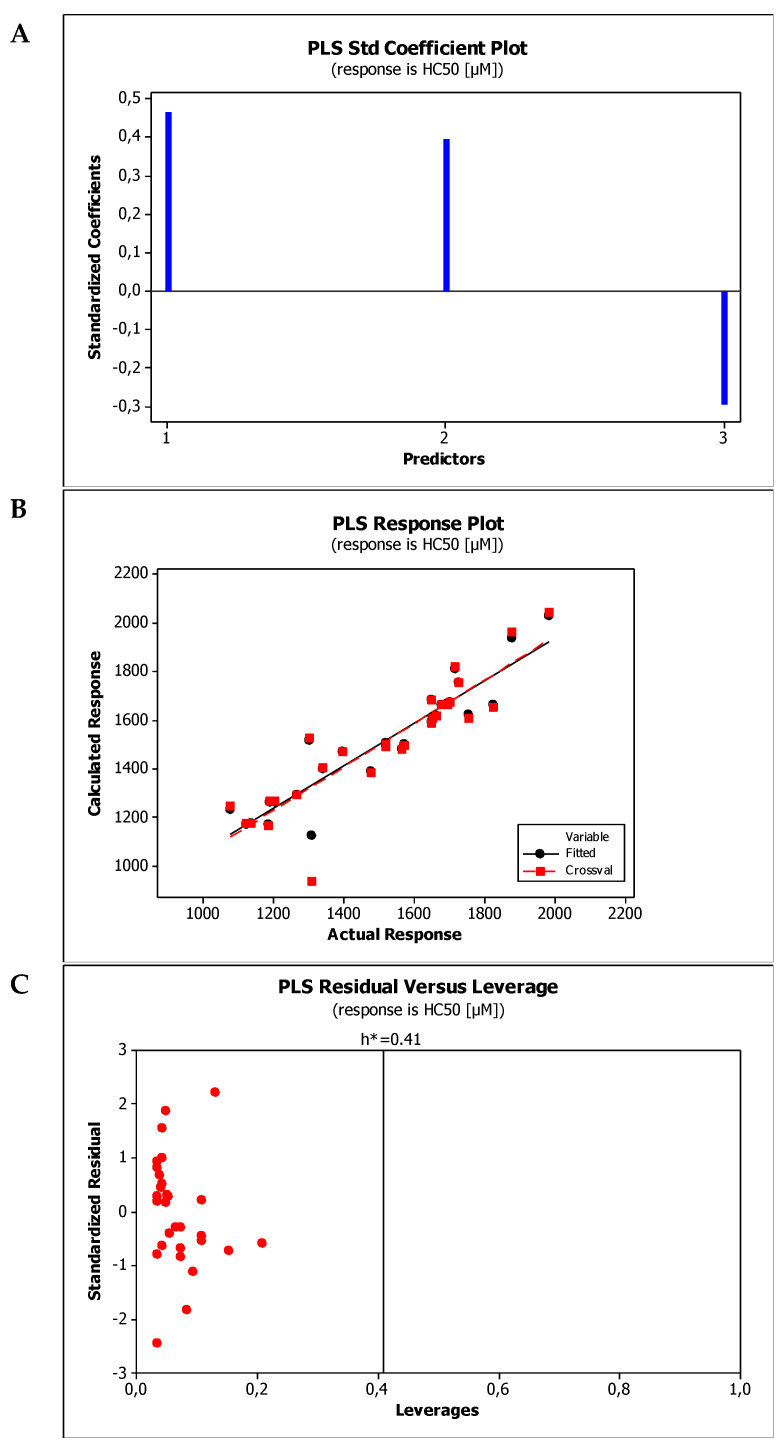
Standardized coefficients (**A**), response plot (**B**), and Williams plot (**C**) obtained for HC_50_ prediction (model M7).

**Figure 5:**
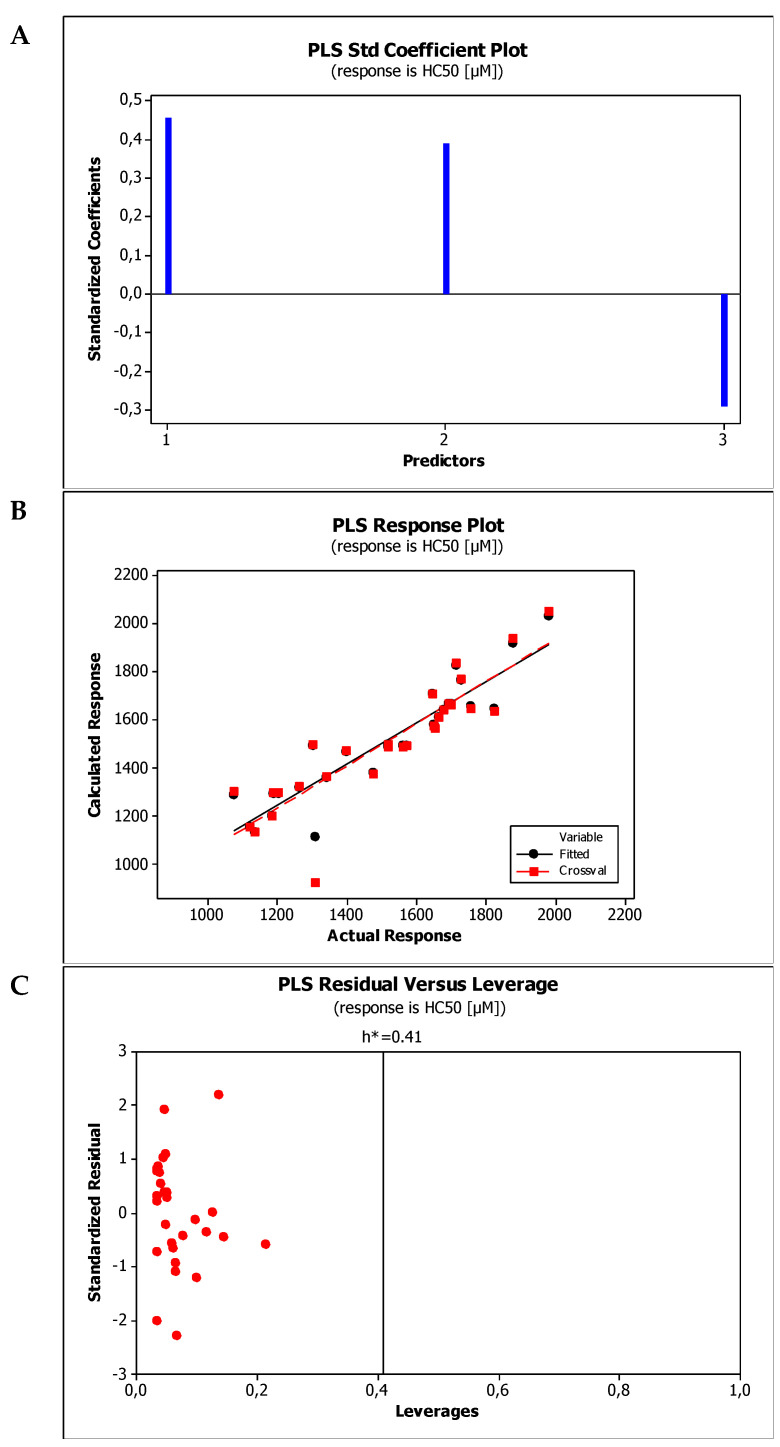
Standardized coefficients (**A**), response plot (**B**), and Williams plot (**C**) obtained for HC_50_ prediction (model M8).

In all cases, the response plots (Figure 2B, Figure 3B, Figure 4B and Figure 5B, and Figures S3B, S4B, S5B and S6B in the Supplementary Material) show very good linear relationships between the values obtained in silico (actual response) and those based on the derived model (calculated response). The Williams plots illustrating the applicability domain (Figure 2C, Figure 3C, Figure 4C and Figure 5C, and Figures S3C, S4C, S5C and S6C in the Supplementary Material), bound by the values ± 3SD and the warning lever h* = 0.41, show that all models are valid within the ranges in which they were developed. The only exception is compound **12** in models M1 (Figure 2C) and M6 (Figure S6C in the Supplementary Material). Despite the comparable statistical evaluation of the derived models, one can try to name the more promising models. In our opinion, these are models M1, M4, and M6, i.e., those which are based on parameters of micellar lipophilicity (log k_BMC_) and allow the prediction of penetration of phenoxyacetic acid-derived congeners through the plant cuticle (model M1) and the skin (model M4) as well as binding to human serum albumin (model M6). These models show slightly more favorable statistics. This is good news, because the use of micellar chromatography in screening tests of potentially bioactive substances allows not only the reduction in research costs due to the significantly lower price of the ODS column compared to the IAM column and the reduction in the use of organic reagents when necessary in the case of more lipophilic molecules, but also compliance with the principles of green chemistry. Exclusively in the case of the prediction of blood–brain barrier permeability (log BB), a valuable model (M3) for the log k_w,IAM_ parameters was derived, i.e., measured on the IAM column.

The robustness and high predictive ability of the derived models are also confirmed by the leave-33%-out cross-validation, presented in the Supplementary Material (Figure S7) as response plots [44].

The derived QSPR models can predict not only important biological properties of the tested congeners, but also indicate those characteristics of the compounds (molecular descriptors) that have a significant influence on a particular property. This is well illustrated by the diagrams in Figure 2A and Figure 3A, and Figures S3A, S4A, S5A and S6A in the Supplementary Material. Using these diagrams, we can assess the influence of the individual molecular descriptors (independent variables in the model) on the individual biological property of the compounds. The derived models show that the penetration of each phenoxyacetic acid congener through the plant cuticle, the skin, and the blood–brain barrier, as well as the binding to human serum albumin depends on three properties of the compound: its lipophilicity, its polarizability, and the sum of the hydrogen bond donors and acceptors in its molecule. As the graphs of the coefficients of the standardized values of log P_w/pc_ (Figure 2A and Figure 3A), log BB (Figure S3A in the Supplementary Material), log K_p_ (Figures S4A and S5A in the Supplementary Material), and log P_w/HSA_ (Figure S6A in the Supplementary Material) show, they increase with an increase in the lipophilicity and polarizability of the molecules, and they decrease with an increase in the number of hydrogen bond donors and acceptors. The positive effect of lipophilicity on the above parameters is completely understandable due to the lipophilic nature of biological membranes—the plant cuticle [45], the skin [46,47] and the blood–brain barrier [48,49,50,51]. Only compounds that are sufficiently lipophilic can overcome these barriers. At the same time, the polarizability of molecules increases their ability to interact with the lipid layer due to van der Waals interactions (inductive and dispersive). Polarizability as a parameter related to the size of the molecule also indirectly indicates the positive effect of the size of the molecule on an increase in its lipophilicity. Of course, these conclusions are limited to the molecules studied in this article, i.e., with a MW of no more than 300 g mol^−1^. Similarly, the lipophilicity and polarizability of molecules influence their interaction with albumins [52]. Increasing the polarity of the molecule, in the case of the derived models described by the sum of the number of hydrogen bond donors and acceptors (HBD+HBA), increases the solubility of the compound in water, decreases its affinity for the lipid phase, and thus reduces the penetration through biological membranes (decreases the log P_w/pc_, log BB and log K_p_ values) and weakens the binding to human serum albumin (decreases the log P_w/HSA_ values). As shown in Figure S3A in the Supplementary Material, this effect is the weakest in the case of the log BB parameter. These results are also consistent with our previous findings [53,54].

As mentioned above, the known phenoxyacetic acid-derived herbicides can have a detrimental effect on erythrocytes and cause hemolysis. As this is an undesirable side effect, it is necessary to predict the potential hazard in the case of their newly evaluated congeners. In our studies, based on the QSARs methodology, we attempted to derive a model combining the experimental HC_50_ values (Table 2) with the molecular descriptors of the tested compounds (Table 3). The application of the procedure described above resulted in two models (M7 and M8 from Table 4) based on the parameters log k_BMC_ (model M7) and log k_w,IAM_ (model M8), whose statistics are presented in Table 5. Both models were validated, and in the statistical evaluation they should be considered very good and highly predictive; the values of the determination coefficients (also cross validated) are high and very good (R^2^, R^2^_adj_ and Q^2^_cv_ > 0.8) and good (R^2^_pred_ > 0.7). Also, the Williams plots indicate that the models are valid within the domains in which they were developed (Figure 4C and Figure 5C). As indicated by the standardized coefficients (Figure 4A and Figure 5A), the HC_50_ values depend on the lipophilicity of the compound, the molecular weight, and the number of hydrogen bond acceptors. This means that the hemolytic effect of the tested phenoxyacetic acid-derived congeners decreases (the HC_50_ value increases) with increasing lipophilicity and molecular size and increases (the HC_50_ value decreases) with increasing the number of hydrogen bond acceptors in the molecule. In the case of the tested herbicide-like molecules, this is synonymous with increasing the number of oxygen atoms. In other words, the tested potential herbicides are more toxic the better they dissolve in water, the smaller their molecules are, and the more hydrogen bond acceptors (oxygen atoms) there are in their molecules. In the case of the tested phenoxyacetic acid-derived congeners, the most toxic are molecules **7**, **8**, **9**, **24**, **25**, and **27**, for which the HC_50_ values range from 1078 to 1205 µM. These compounds simultaneously meet the conditions indicated above: low molar mass (from 168.15 g mol^−1^ to 182.17 g mol^−1^), high HBA value = 4, and weak lipophilicity. Both the in silico (log P_o/w_) and the in vitro chromatographic descriptors (log k_BMC_ and log k_w,IAM_) assess these congeners as weakly lipophilic (Table 3).

The results obtained indicate that the least lipophilic compounds **24** and **25**, which are unable to penetrate the plant cuticle, should not be considered as potential herbicides and as such be excluded from further testing.

## 3. Materials and Methods

### 3.1. Herbicides and Herbicide-like Compounds

**Scheme 1:**
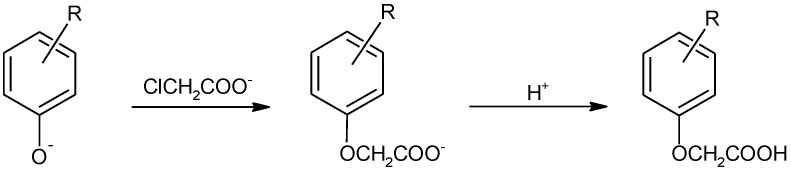
The synthesis of **1** (R = 4-CH_3_), **9** (R = 4-OCH_3_), and **19** (R = 2,4-Cl).

2-(4-Methylphenoxy)acetic acid (**1**), 2-(4-methoxyphenoxy)acetic acid (**9**), and 2-(2,4-dichlorophenoxy)acetic acid (2,4-D) (**19**) were obtained in a two-step synthetic approach by condensing sodium *para*-metylphenolate or sodium *para*-methoxyphenolate or sodium 2,4-dichlorophenolate with sodium chloroacetate, followed by treatment with hydrochloric acid, according to the published procedures [55,56,57]. As a result, a nucleophilic displacement between the appropriate phenolate and chloroacetate anions could be realized (Scheme 1).

After recrystallization of the crude reaction products, the pure samples of compounds **1**, **9**, and **19** were identified based on their melting points (142–143 °C, 109–113 °C, 134–136 °C, respectively), identical to those reported in the literature [56,57]. Other herbicides and herbicide-like molecules bought in Chemat (Gdańsk, Poland) were of the highest grade available. Three commercially approved herbicides (**17**, **19**, and **20**) have previously been utilized by our research team as electrophilic reagents suitable for the synthesis of potential anticancer and antimicrobial agents [58].

### 3.2. Hemolysis Assay

The hemolytic properties of commercially used herbicides such as CMPA (**17**), 2,4-D (**19**), 2,4,5-T (**20**), and the remaining herbicide-like molecules (**1**–**16**, **18**, **21**–**29**) were determined using an ex vivo model of erythrocytes. Defibrinated sheep blood purchased from Biomaxima (Lublin, Poland) was used in the hemolysis assay. Briefly, the blood was centrifuged at 1500 rpm for 10 min, the plasma was removed, and the cells were washed three times with a phosphate-buffer saline (PBS, pH 7.4; Biomed, Lublin, Poland). A 4% suspension of red blood cells in PBS was used for further procedures. The erythrocyte suspension was incubated with a solution of each herbicide or herbicide-like compound at one of the ten concentrations tested (250, 500, 750, 1000, 1250, 1500, 1750, 2000, 3000, and 4000 µM) for 1 h at 37 °C. Red blood cells incubated with PBS were the negative control, and those with triton X-100 (polyethylene glycol *tert*-octylphenyl ether solution; Sigma-Aldrich, Saint Louis, MO, USA) served as the positive control. Next, the samples were centrifuged at 3000 rpm for 10 min, and the absorbance of supernatants was measured at 540 nm using a Hitachi U2800 UV-Vis spectrophotometer (Tokyo, Japan). The degree of hemolysis was calculated according to the following equation:

(9) $$Hemolysis\left(\%\right)=\frac{{Abs}_{sample}-{Abs}_{blank}}{{Abs}_{control}-{Abs}_{blank}}\times 100\%$$

where Abs_sample_ is the absorbance of the tested compound, Abs_blank_ is the absorbance of phosphate-buffered saline, and Abs_control_ is the absorbance of triton X-100.

Based on the obtained results, the HC_50_ values (the lowest concentration of the herbicide or herbicide-like compound causing 50% hemolysis during the experiment) were calculated (using the probit method [59]) for each tested congener. At all stages of the procedure, the samples were thoroughly mixed. All experiments were performed in triplicate under similar conditions.

### 3.3. Chromatographic Measurements

The LC-10 AT liquid chromatograph (Shimadzu, Kyoto, Japan) used for the measurements consists of the Rheodyne dosing valve with a 20 μL loop, the LC-10AT pump, the DGU 14A degasser, the SPD-10A UV-Vis detector, the SCL-10A control system, and the CTO 10AS thermostat. The stationary phases used were the columns IAM.DD2, 100 × 4.6 mm and 10 μm particle size (Regis Chemicals, Morton Grove, IL, USA) and Spherisorb ODS-3, 125 × 4 mm and 5 μm particle size (Merck, Lublin, Poland). A buffer with a pH value of 7.4 was used as the mobile phase for the IAM.DD2 column. The buffer was prepared from 0.01 M disodium hydrogen phosphate and 0.02 M citric acid. For the ODS-3 column, a 0.04 M solution of the surfactant Brij 35 (2-(dodecyloxy)ethan-1-ol) in the same buffer was used as the mobile phase.

The retention times (t_r_) were measured at 25 °C with an eluent flow rate of 1 mL min^−1^. All the phenoxyacetic acid-derived congeners that were chromatographed were detected under UV-Vis light at λ = 254 nm. The dead time (t_0_) was determined using the citric acid peak. The test compounds were dissolved in acetonitrile. The measurements were carried out in triplicate. The values of the retention factors were calculated using the following formula:

log k = log((t_r_ − t_0_)/t_0_) (10)

### 3.4. In Silico Calculations

Molecular descriptors (MW—molar weight; TPSA—topological polar surface area; α—polarizability; HBD—hydrogen bond donors; HBA–hydrogen bond acceptors; NRB–the number of rotatable bonds), pK_a_ (K_a_—the acid dissociation constant), and biological (log P_o/w_, log P_w/pc_, log K_p_, log P_w/HSA_, log BB) parameters of the tested compounds were calculated according to 2-D molecular structures of compounds using ACD/Percepta software, version 1994–2012 (ACD/Labs, Advanced Chemistry Development, Inc., Toronto, ON, Canada).

## 4. Conclusions

Using a hybrid procedure, combining in vitro biomimetic liquid chromatographic techniques (IAM and BMC) and in silico QSARs, the models were derived to predict selected biological properties such as the penetration through the plant cuticle (log P_w/pc_), the skin (log K_p_), and the blood–brain barrier (log BB), as well as the binding to human serum albumin (log P_w/HSA_) of phenoxyacetic acid-derived congeners evaluated as potential herbicides. Reliable and high-concept models were developed, indicating the lipophilicity, polarizability, and sum of hydrogen bond donors and acceptors as properties determining the biological effectiveness of these substances. Biopartitioning micellar chromatography was indicated as the technique of choice for predicting the log P_w/pc_, log K_p_, and log P_w/HSA_ values, while IAM chromatography allowed the prediction of log BB values. The results obtained indicate that molecules being more lipophilic, with higher polarizability permeate better through biological membranes (the plant cuticle, skin, and blood–brain barrier), and bind more strongly to human serum albumin. Modeling the toxicity of phenoxyacetic acid-derived congeners to erythrocytes, expressed by the experimental HC_50_ values, indicated that the potential herbicides tested are more toxic the better they dissolve in water, the smaller their molecules are, and the more hydrogen bond acceptors are found in their molecules. The studies demonstrate the effectiveness of chromatographic biomimetic techniques in screening potentially active molecule classes.

## Figures and Tables

**Table 1:** Structures and chemical names for phenoxyacetic acid-derived congeners **1**–**29**.

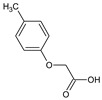 2-(4-Methylphenoxy)acetic acid (**1**)	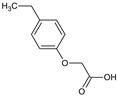 2-(4-Ethylphenoxy)acetic acid (**2**)		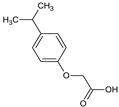 2-(4-Isopropylphenoxy)acetic acid (**3**)
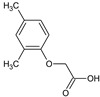 2-(2,4-Dimethylphenoxy)acetic acid (**4**)	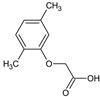 2-(2,5-Dimethylphenoxy)acetic acid (**5**)		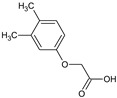 2-(3,4-Dimethylphenoxy)acetic acid (**6**)
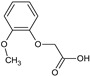 2-(2-Methoxyphenoxy)acetic acid (**7**)	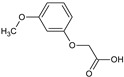 2-(3-Methoxyphenoxy)acetic acid (**8**)		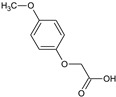 2-(4-Methoxyphenoxy)acetic acid (**9**)
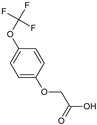 2-[4-(Trifluoromethoxy)phenoxy]acetic acid (**10**)	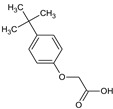 2-(4-*Tert*-butylphenoxy)acetic acid (**11**)		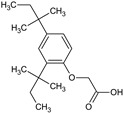 2-(2,4-Di-*tert*-pentylphenoxy)acetic acid (**12**)
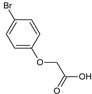 2-(4-Bromophenoxy)acetic acid (**13**)	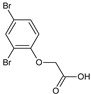 2-(2,4-Dibromophenoxy)acetic acid (**14**)		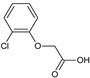 2-(2-Chlorophenoxy)acetic acid (**15**)
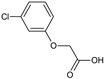 2-(3-Chlorophenoxy)acetic acid (**16**)	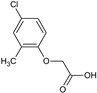 2-(4-Chloro-2-methylphenoxy)acetic acid, CMPA (**17**)		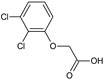 2-(2,3-Dichlorophenoxy)acetic acid (**18**)
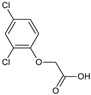 2-(2,4-Dichlorophenoxy)acetic acid, 2,4-D (**19**)	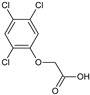 2-(2,4,5-Trichlorophenoxy)acetic acid, 2,4,5-T (**20**)		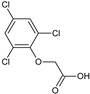 2-(2,4,6-Trichlorophenoxy)acetic acid (**21**)
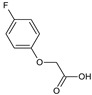 2-(4-Fluorophenoxy)acetic acid (**22**)	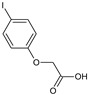 2-(4-Iodophenoxy)acetic acid (**23**)		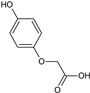 2-(4-Hydroxyphenoxy)acetic acid (**24**)
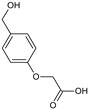 2-[4-(Hydroxymethyl)phenoxy]acetic acid (**25**)	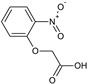 2-(2-Nitrophenoxy)acetic acid (**26**)		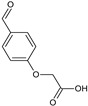 2-(4-Formylphenoxy)acetic acid (**27**)
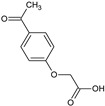 2-(4-Acetylphenoxy)acetic acid (**28**)		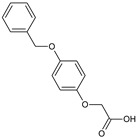 2-[4-(Benzyloxy)phenoxy]acetic acid (**29**)	

**Table 2:** The experimentally determined HC_50_ values for phenoxyacetic acid-derived congeners **1**–**29**.

No.	HC_50_ (µM)	No.	HC_50_ (µM)	No.	HC_50_ (µM)
**1**	1477 ± 32.5	**11**	1826 ± 12.2	**21**	1728 ± 27.1
**2**	1572 ± 88.7	**12**	1982 ± 53.6	**22**	1342 ± 42.0
**3**	1650 ± 31.4	**13**	1678 ± 54.7	**23**	1757 ± 48.6
**4**	1520 ± 85.8	**14**	1878 ± 25.5	**24**	1136 ± 41.8
**5**	1520 ± 93.6	**15**	1398 ± 96.6	**25**	1124 ± 11.0
**6**	1564 ± 46.1	**16**	1302 ± 47.6	**26**	1309 ± 50.1
**7**	1078 ± 20.8	**17**	1665 ± 35.1	**27**	1188 ± 55.3
**8**	1205 ± 15.9	**18**	1695 ± 45.8	**28**	1266 ± 41.4
**9**	1190 ± 30.4	**19**	1701 ± 38.0	**29**	1649 ± 80.7
**10**	1654 ± 39.2	**20**	1716 ± 27.7		

HC_50_—the lowest concentration of the compound causing 50% hemolysis during the experiment. Data (from three independent experiments under similar conditions) are provided as the mean ± standard deviation.

**Table 3:** Molecular descriptors (MW–molar weight; TPSA–topological polar surface area, α–polarizability; HBD–hydrogen bond donors; HBA–hydrogen bond acceptors; NRB–the number of rotatable bonds), pK_a_ (K_a_—the acid dissociation constant), biological (log P_o/w_, log P_w/pc_, log K_p_, log P_w/HSA,_ log BB) and chromatographic (log k_BMC_, log k_w,IAM_) parameters of the tested compounds.

No.	MW [g mol^−1^]	TPSA [Å^2^]	α [Å^3^]	HBD	HBA	NRB	pK_a_	log P_o/w_	log P_w/pc_	log K_p_	log P_w/HSA_	log BB	log k_BMC_	log k_w,IAM_
**1**	166.17	46.53	17.42	1	3	3	3.22	1.853	1.559	−5.399	−0.177	−0.244	0.234	−0.363
**2**	180.20	46.53	19.30	1	3	3	3.21	2.390	2.110	−5.075	0.051	−0.118	0.572	0.156
**3**	194.23	46.53	21.14	1	3	3	3.21	2.805	2.548	−4.838	0.232	0.001	0.817	0.486
**4**	180.20	46.53	19.34	1	3	4	3.27	2.470	2.153	−5.050	0.097	−0.052	0.583	0.178
**5**	180.20	46.53	19.34	1	3	4	3.23	2.470	2.153	−5.050	0.097	−0.052	0.543	0.120
**6**	180.20	46.53	19.34	1	3	4	3.23	2.470	2.153	−5.050	0.097	−0.052	0.489	0.134
**7**	182.17	55.76	18.16	1	4	5	3.21	0.684	0.563	−6.284	−0.735	−0.830	−0.160	−0.526
**8**	182.17	55.76	18.16	1	4	4	3.14	1.387	1.087	−5.812	−0.431	−0.417	−0.030	−0.508
**9**	182.17	55.76	18.16	1	4	7	3.24	1.183	0.919	−5.953	−0.508	−0.470	−0.030	−0.508
**10**	236.14	55.76	18.34	1	4	3	3.05	1.742	1.432	−5.383	−0.440	−0.367	0.937	0.466
**11**	208.25	46.53	22.80	1	3	3	3.22	3.379	3.060	−4.518	0.434	0.149	0.981	0.700
**12**	292.41	46.53	33.76	1	3	3	3.27	6.535	6.201	−2.679	1.741	0.956	1.670	1.850
**13**	231.04	46.53	18.56	1	3	3	3.09	1.928	1.815	−5.441	0.062	−0.277	0.721	0.302
**14**	309.94	46.53	21.61	1	3	3	2.98	2.843	2.897	−4.931	0.667	−0.064	1.070	0.827
**15**	186.59	46.53	17.45	1	3	3	3.07	1.760	1.530	−5.488	−0.156	−0.327	0.351	−0.137
**16**	186.59	46.53	17.45	1	3	3	3.08	1.920	1.743	−5.328	−0.050	−0.274	0.567	0.024
**17**	200.62	46.53	19.37	1	3	3	3.14	2.323	2.123	−5.156	0.124	−0.152	0.872	0.598
**18**	221.04	46.53	19.39	1	3	3	2.96	2.457	2.331	−5.042	0.245	−0.177	0.856	0.620
**19**	221.04	46.53	19.39	1	3	3	2.98	2.413	2.335	−5.053	0.254	−0.184	0.892	0.608
**20**	255.48	46.53	21.33	1	3	3	2.88	3.118	3.171	−4.592	0.673	−0.048	1.114	1.094
**21**	255.48	46.53	21.33	1	3	3	2.87	3.007	3.022	−4.704	0.609	−0.055	0.855	0.715
**22**	170.14	46.53	15.51	1	3	4	3.13	1.274	1.038	−5.665	−0.457	−0.437	0.222	−0.554
**23**	278.04	46.53	20.63	1	3	4	3.08	2.287	2.218	−5.372	0.390	−0.123	0.011	−0.376
**24**	168.15	66.76	16.26	2	4	4	3.27	0.424	-0.080	−6.768	−0.668	−0.956	−0.274	−1.298
**25**	182.17	66.76	18.07	2	4	4	3.15	0.582	0.000	−6.758	−0.726	−0.735	−0.443	−1.393
**26**	197.14	95.36	18.11	1	6	6	2.91	1.121	1.173	−5.942	−0.338	−0.704	0.153	−0.502
**27**	180.16	63.60	18.19	1	4	3	3.04	0.459	0.343	−6.517	−0.733	−0.804	−0.422	−1.079
**28**	194.18	63.60	19.49	1	4	3	3.01	1.014	0.824	−6.223	−0.538	−0.649	−0.041	−0.526
**29**	258.27	55.76	27.87	1	4	3	3.24	3.020	3.077	−5.112	0.606	−0.125	1.002	0.996

**Table 4:** The established QSAR models.

Model No.	Model
M1	log P_w/pc_ = −0.809(±0.510) + 0.953(±0.179)log k_BMC_ + 0.191(±0.022)α − 0.339(±0.091)(HBD + HBA)
M2	log P_w/pc_ = −0.149(±0.540) + 0.747(±0.151)log k_w,IAM_ + 0.170(±0.027)α − 0.304(±0.100)(HBD + HBA)
M3	log BB = −0.385(±0.241) + 0.131(±0.068)log k_w,IAM_ + 0.050(±0.011)α − 0.198(±0.045)(HBD + HBA)
M4	log K_p_ = −6.121(±0.438) + 0.764(±0.153)log k_BMC_ + 0.084(±0.019)α − 0.283(±0.078)(HBD + HBA)
M5	log K_p_ = −5.610(±0.478) + 0.572(±0.134)log k_w,IAM_ + 0.071(±0.024)α − 0.267(±0.086)(HBD + HBA)
M6	log P_w/HSA_ = −0.8375(±0.336) + 0.308(±0.094)log k_BMC_ + 0.076(±0.016)α − 0.150(±0.062)(HBD + HBA)
M7	HC_50_ = 1189.68(±141.15) + 263.29(±48.84)log k_BMC_ + 2.27(±0.57)MW − 82.71(±29.30)HBA
M8	HC_50_ = 1351.34(±157.04) + 179.72 (±38.62)log k_w,IAM_ + 2.14(±0.66)MW − 88.82(±31.46)HBA

Numerical values in parenthesis denote standard deviations.

**Table 5:** Statistics of the established QSPR models.

Model No.	R^2^	R^2^_adj_	R^2^_pred_	PRESS	VIF *	SS	MSE	SD	Q^2^_cv_	PRESS_cv_
M1	0.9428	0.9419	0.8692	5.56762	<2.8	40.3664	0.0883	0.2971	0.9481	5.56762
M2	0.9439	0.9372	0.8514	6.32727	<3.8	40.1859	0.0955	0.3090	0.9439	6.32727
M3	0.8765	0.8617	0.7433	0.98749	<3.8	3.37173	0.01900	0.1378	0.8765	0.98750
M4	0.9094	0.8985	0.7658	4.19807	<2.8	16.3026	0.06498	0.2549	0.9094	4.19807
M5	0.8956	0.8831	0.7126	5.15174	<3.8	16.0554	0.07487	0.2736	0.8956	5.15174
M6	0.8924	0.8795	0.8066	1.66068	<3.8	7.66453	0.03697	0.1923	0.8975	1.62810
M7	0.8818	0.8676	0.7487	441,915	<2.2	1,550,450	8312	91.1712	0.8818	441,915
M8	0.8630	0.8466	0.7371	462,177	<2.5	240,817	9633	98.1462	0.8630	462,177

The coefficient of determination (R^2^, Q^2^), the determination coefficient adjusted (R^2^_adj_), the determination coefficient predicted (R^2^_pred_), the predicted residual error sum of squares (PRESS), the variance inflation factor (VIF), the sum of squared differences from the mean (SS), the mean squared error (MSE), standard deviation (SD); *—the highest value; cv—cross validated.

## Data Availability

Data are contained within the article and Supplementary Material.

## Supplementary Materials

The following supporting information can be downloaded at: https://www.mdpi.com/article/10.3390/molecules30030688/s1, Figure S1. Hemolytic activities of the tested phenoxyacetic acid-derived congeners (**1**–**29**). Figure S2. An exemplary graph showing % hemolysis vs. concentration (for compound **26**). Figure S3: Standardized coefficients (A), response plot (B), and Williams plot (C) obtained for log BB prediction (model M3). Figure S4: Standardized coefficients (A), response plot (B), and Williams plot (C) obtained for log K_p_ prediction (model M4). Figure S5: Standardized coefficients (A), response plot (B), and Williams plot (C) obtained for log K_p_ prediction (model M5). Figure S6: Standardized coefficients (A), response plot (B), and Williams plot (C) obtained for log P_w/HSA_ prediction (model M6). Figure S7: Response plots obtained for leave-33%-out cross validation of the derived models M1–M8.
