# Supplementary material for: Biomimetic Chromatography/QSAR Investigations in Modeling Properties Influencing the Biological Efficacy of Phenoxyacetic Acid-Derived Congeners
Source: Molecules. 2025 Feb 4;30(3):688. doi: 10.3390/molecules30030688 (PMC11819946; doi:10.3390/molecules30030688)
Supplement: Supplementary file 1 [file molecules-30-00688-s001.zip › molecules-3418779-supplementary.pdf]

Supplementary Material

Biomimetic Chromatography/QSAR Investigations in Modeling Properties Influencing the Biological Efficacy of Phenoxyacetic Acid-Derived Congeners

Małgorzata Janicka [1], Małgorzata Sztanke [2] and Krzysztof Sztanke [3,*]

[1] Department of Physical Chemistry, Faculty of Chemistry, Institute of Chemical Science, Maria Curie-Skłodowska University, 20-031 Lublin, Maria Curie-Skłodowska Sq. 2, Poland, malgorzata.janicka@mail.umcs.pl

[2] Department of Medical Chemistry, Medical University of Lublin, 4A Chodźki Street, 20-093 Lublin, Poland, malgorzata.sztanke@umlub.pl

[3] Laboratory of Bioorganic Compounds Synthesis and Analysis, Medical University of Lublin, 4A Chodźki Street, 20-093 Lublin, Poland

* Correspondence: krzysztof.sztanke@umlub.pl

Figure S1. Hemolytic activities of the tested phenoxyacetic acid-derived congeners (**1-29**)………………………………………………………………………… 2

Figure S2. An exemplary graph showing % hemolysis vs. concentration (for compound **26**)……………………………………………………………………….. 2

Figure S3. Standardized coefficients (A), response plot (B), and Williams plot (C) obtained for log BB prediction (model M3)……………………………………… 3

Figure S4. Standardized coefficients (A), response plot (B), and Williams plot (C) obtained for log K_P prediction (model M4)….………………………………… 4

Figure S5. Standardized coefficients (A), response plot (B), and Williams plot (C) obtained for log K_P prediction (model M5)……………………………………… 5

Figure S6. Standardized coefficients (A), response plot (B), and Williams plot (C) obtained for log $P_{w/HSA}$ prediction (model M6)………………………………… 6

Figure S7. Response plots obtained for leave-33%-out cross validation of the derived models M1-M8………………………………………………………………... 7

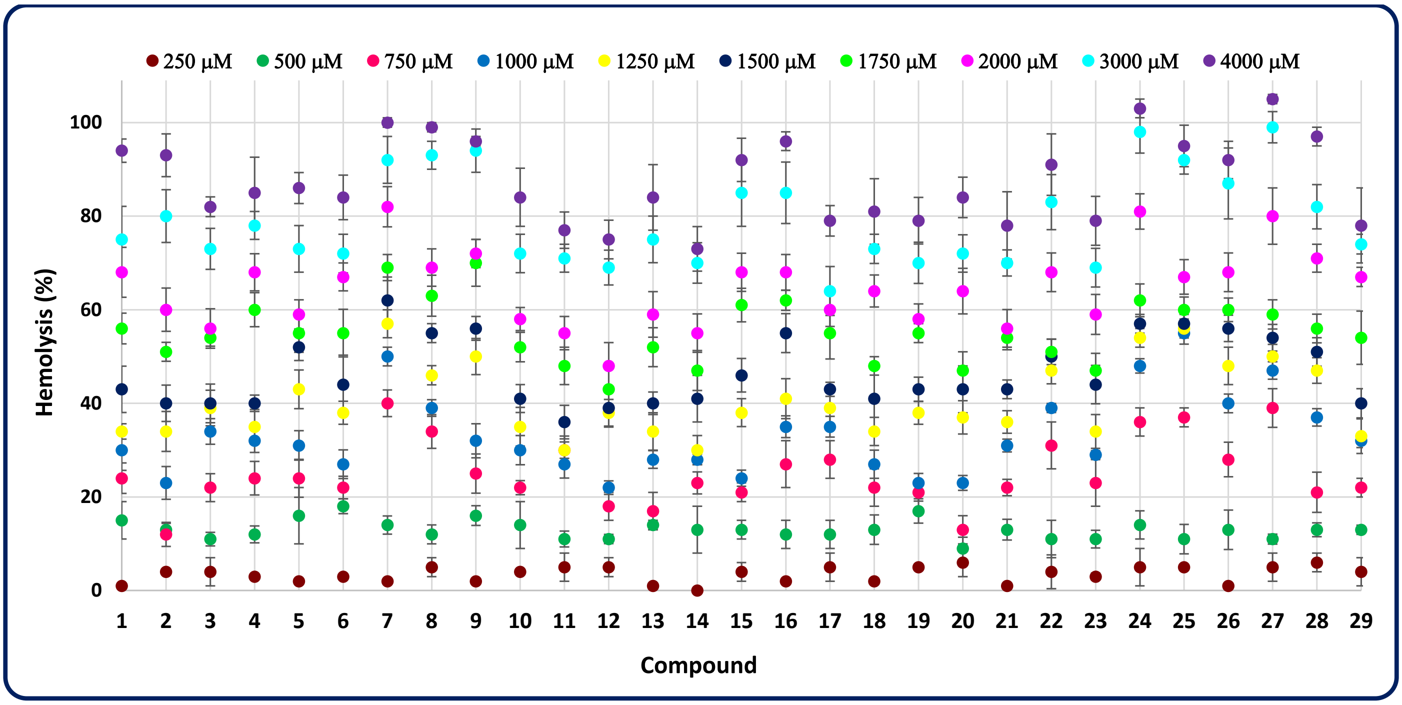

Figure S1. Hemolytic activities of the tested phenoxyacetic acid-derived congeners (**1-29**).

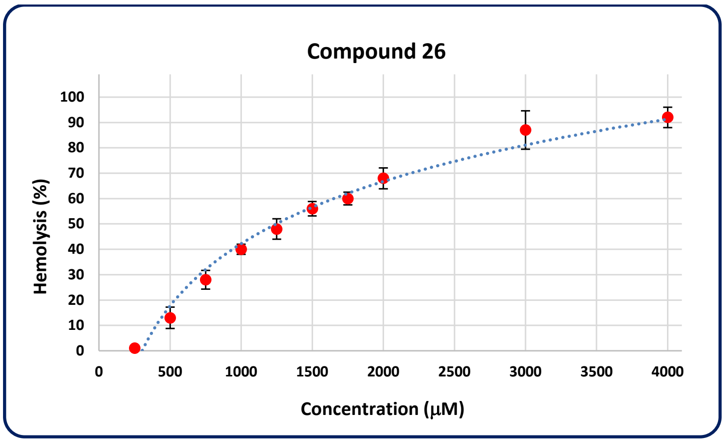

Figure S2. An exemplary graph showing % hemolysis vs. concentration (for compound **26**).

A

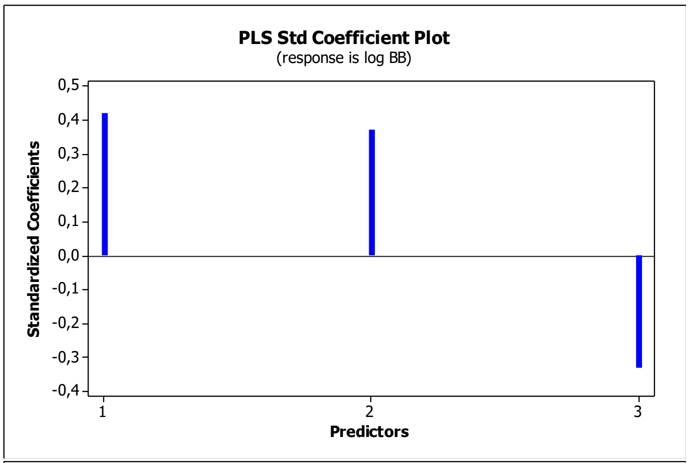

B

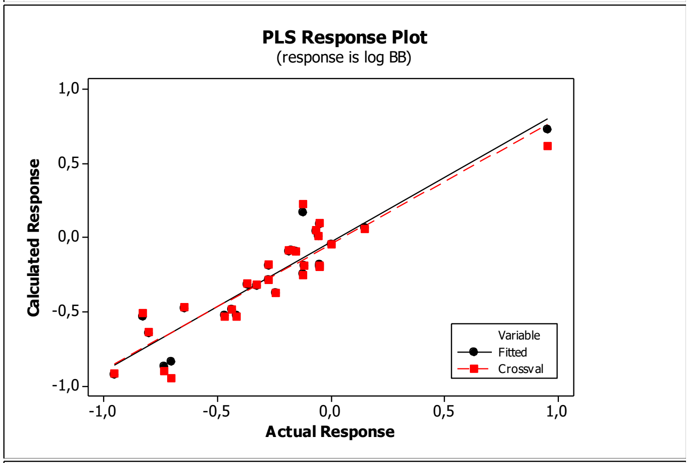

C

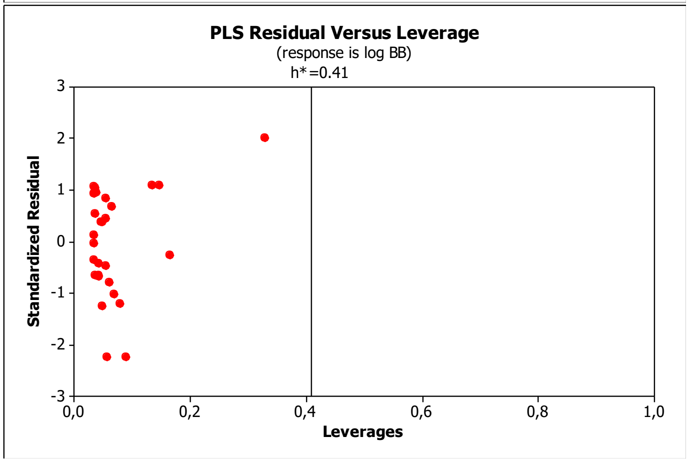

Figure S3. Standardized coefficients (**A**), response plot (**B**), and Williams plot (**C**) obtained for log BB prediction (model M3).

A

B

C

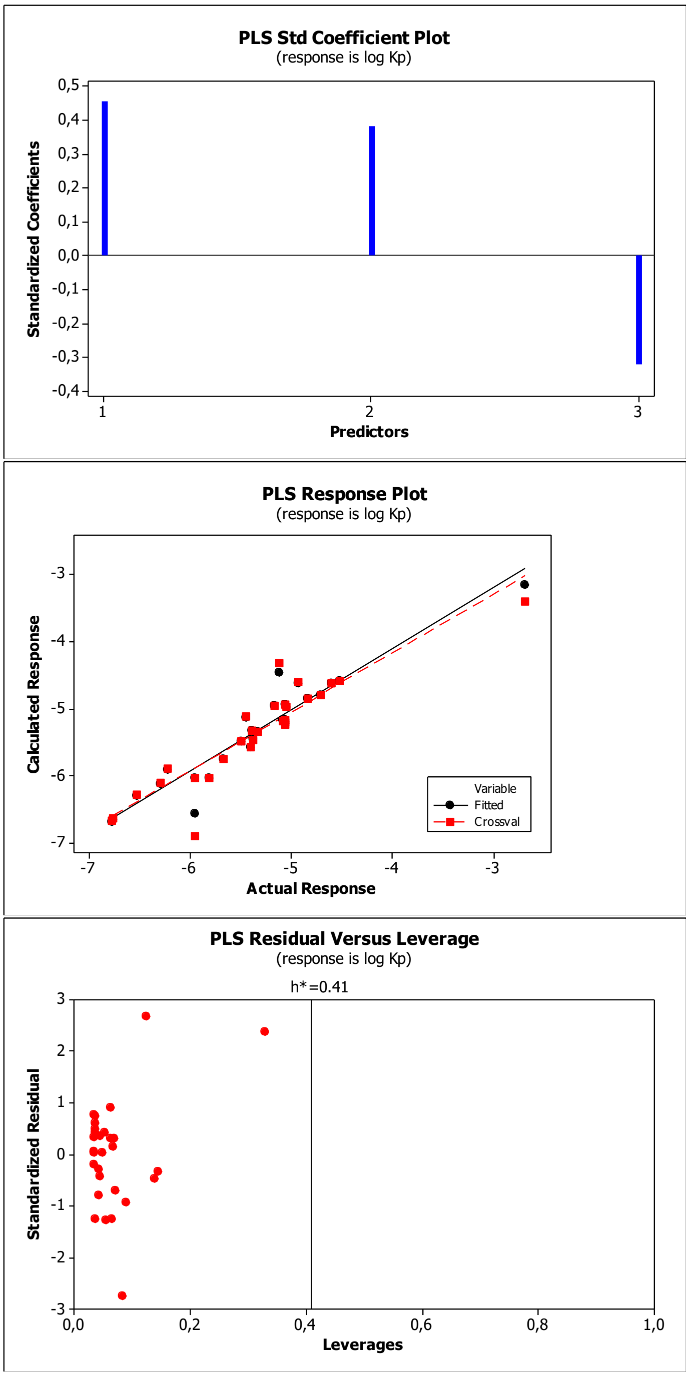

Figure S4. Standardized coefficients (**A**), response plot (**B**), and Williams plot (**C**) obtained for log K_P prediction (model M4).

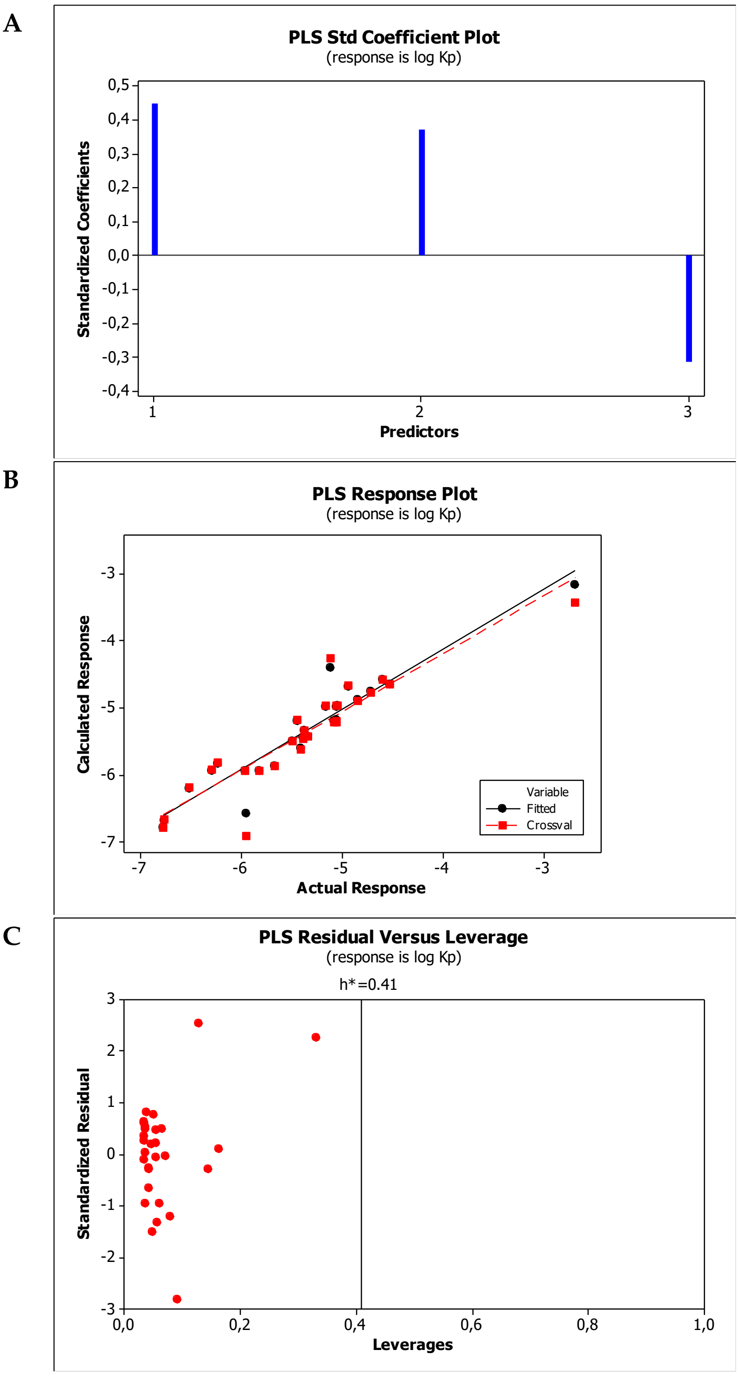

Figure S5. Standardized coefficients (**A**), response plot (**B**), and Williams plot (**C**) obtained for log K_p prediction (model M5).

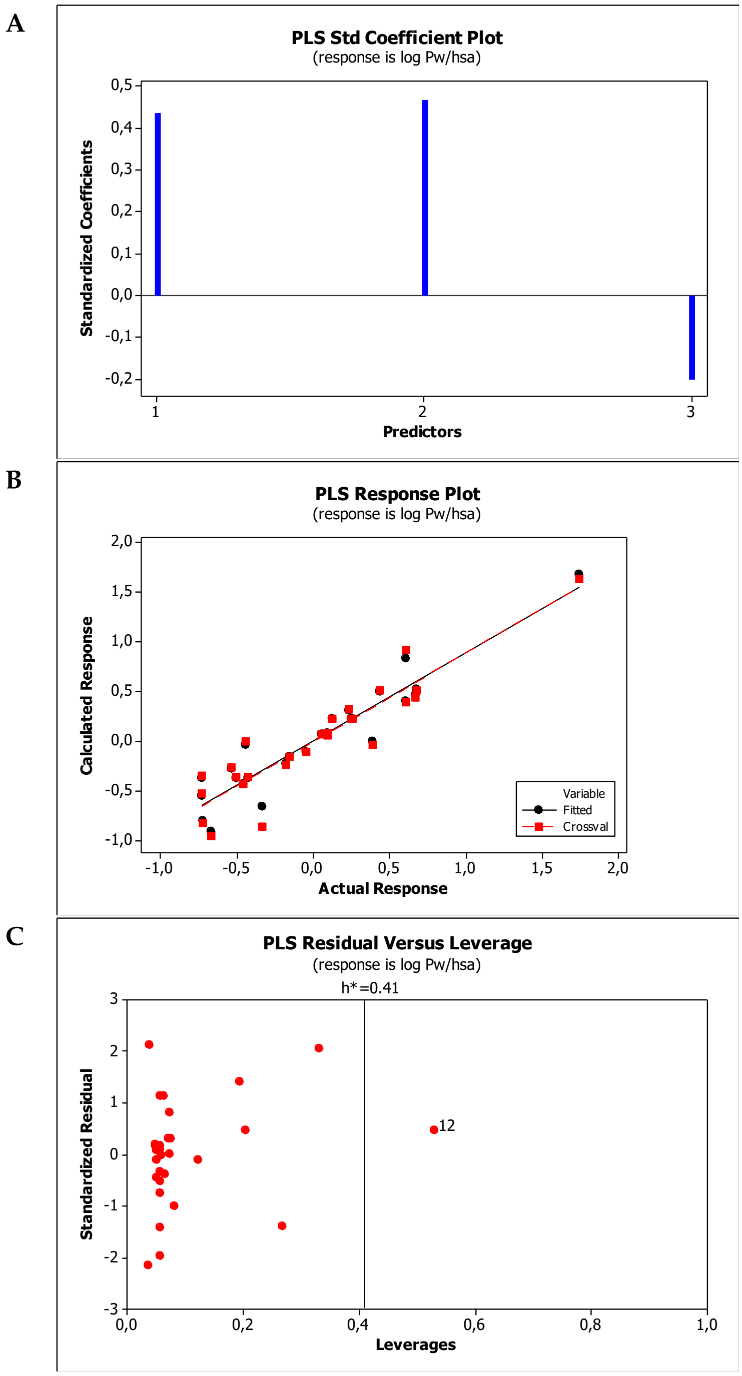

Figure S6. Standardized coefficients (**A**), response plot (**B**), and Williams plot (**C**) obtained for log $P_{w/HSA}$ prediction (model M6).

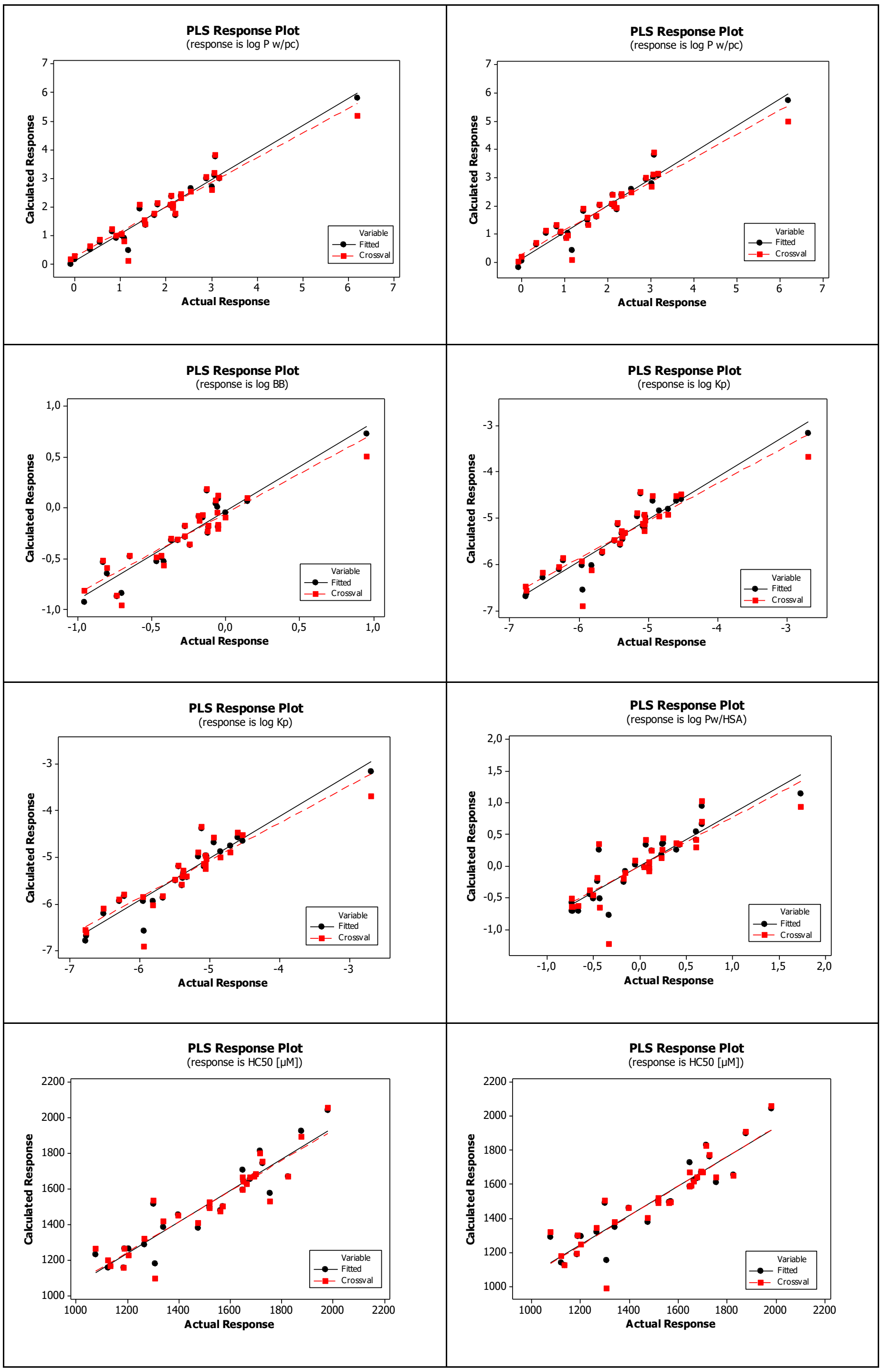

Figure S7. Response plots obtained for leave-33%-out cross validation of the derived models M1-M8.
